# Reply: Hereditary myopathy with early respiratory failure is caused by mutations in the titin FN3 119 domain

**DOI:** 10.1093/brain/awt306

**Published:** 2013-11-21

**Authors:** Gerald Pfeffer, Helen Griffin, Angela Pyle, Rita Horvath, Patrick F. Chinnery

**Affiliations:** 1 Institute of Genetic Medicine, Newcastle, NE1 3BZ, UK; 2 Department of Neurology, Royal Victoria Infirmary, Newcastle, NE1 4LP, UK

Sir, The letter from Hedberg *et al.* (2013) is of great interest because it addresses an important question relating to the genetic aetiology of hereditary myopathy with early respiratory failure (HMERF). The original report by Lange *et al.* (2005) indicated that HMERF was associated with a heterozygous g.296459C>T/p.R32450W mutation in the kinase domain of titin (*TTN*) (using Genebank AJ277892 and Uniprot Q8WZ42 as the reference sequences). Since then, no further cases of HMERF caused by kinase domain mutations in *TTN* have been reported. In contrast, only a year since the report of a mutation in the 119th fibronectin-3 (FN3) domain of *TTN* causing HMERF (Ohlsson *et al.*, 2012; Pfeffer *et al.*, 2012), numerous reports have confirmed an association between the g.274375T>C/p.C30071R FN3 domain mutation and this disease, and seven other mutations have been reported affecting the same domain of titin (Izumi *et al.*, 2013; Palmio *et al.*, 2013; Pfeffer *et al.*, 2013; Toro *et al.*, 2013). Furthermore, our recent study screening 127 patients with myofibrillar myopathy for mutations in *TTN* revealed seven families with mutations in the 119th FN3 domain, but none with kinase domain mutations (Pfeffer *et al.*, 2013).

Ostensibly, the patients from Lange *et al.*’s (2005) original report were from three separate families, although they all shared the same *TTN* haplotype (Lange *et al.*, 2005). Therefore, for all intents and purposes, the kinase domain variant has still only been reported in a single HMERF family. Hedberg *et al.* (2013) now report that patients from the same family described by Lange *et al.* (2005) not only carry the kinase domain mutation (g.296459C>T/p.R32450W), but also have a g.284762C>T mutation in the 119th fibronectin-3 domain, predicted to cause p.P30091L, which is known to cause HMERF on its own (Palmio *et al.*, 2013; Pfeffer *et al.*, 2013). This finding should be confirmed by the authors of the study by Lange *et al.* (2005), but assuming that Hedberg *et al.*’s (2013) report applies to all family members, these data suggest that the g.284762C>T/p.P30091L FN3 domain variant is the true cause of HMERF in the original study of Lange *et al.* (2005). The segregation of both the FN3 and kinase variants with the disease in these patients is likely because of the shared *TTN* haplotype between the patients.

It should be noted that Lange *et al.* (2005) presented functional data in support of the kinase variant’s pathogenicity by creating a recombinant *TTN* kinase construct using previously published methods (Mayans *et al.*, 1998). The catalytic activity of the mutated construct was similar to wild-type, although binding with NBR1 was reduced. This altered NBR1 binding was postulated to be the disease-causing mechanism for this variant. However, the construct included only 324 amino acids from this giant protein, whose inferred complete model includes up to 34 350 amino acids (Uniprot Q8WZ42). We therefore do not know whether this kinase domain variant affects the full-length protein, nor whether the altered NBR1 binding of this particular domain is, in itself, pathogenic.

To determine whether kinase domain variants contribute to the pathogenesis, we sequenced the entire kinase domain (according to Uniprot Q8WZ42, including positions p.32115–32496) in 33 HMERF patients from eight families with the g.274375T>C/p.C30071R mutation in the 119th FN3 domain of *TTN* (Pfeffer *et al.*, 2012, 2013). Our hypothesis was that amino acid sequence-altering variants (previously reported, or novel) would be more prevalent in patients with HMERF than control subjects, and/or that kinase variants would be present in HMERF patients with more severe phenotype. Our control group consisted of 343 disease controls from 261 pedigrees (70% of whom originate from the UK) who have had their exomes sequenced at our centre.

PCR was performed with Immolase™ (Bioline) according to manufacturer’s protocol, using ∼50 ng of DNA, 0.25 mM of each oligonucleotide and 2 mM MgCl_2_, for 30 cycles with annealing temperature of 60°C. Sequencing was performed using BigDye® (Applied Biosystems) according to the manufacturer’s protocol with an ABI 3130XL sequencer. Primer sequences are available on request.

One HMERF patient (Patient A-III:7 from Pfeffer *et al.*, 2012) with the g.274375T>C/p.C30071R mutation had a protein-altering variant, g.295853G>A predicted to cause p.V32248I in the kinase domain (listed in dbSNP as rs34924609—note this is not the same kinase variant as the report from Lange *et al.*, 2005). No sequence-altering variants were found in the other 32 patients. Given that all 33 share the same *TTN* haplotype (Pfeffer *et al.*, 2013), the g.295853G>A/p.V32248I kinase variant in Patient A-III:7 is likely to be in *trans* with the FN3 domain mutation. This patient did not have distinguishing phenotypic features compared with other family members: she had proximal-predominant skeletal muscle weakness, respiratory muscle weakness with abnormal pulmonary function testing, and disease onset occurred at age 55 (onset age ranged from 33 to 71 years in this family).

Comparison of sequence variants in our control group of 343 individuals identified a sibling pair with the g.296459C>T/p.R32450W mutation in the kinase domain (i.e. the variant associated with HMERF in Lange *et al.*, 2005). These patients have a confirmed diagnosis of an autosomal recessive limb girdle muscular dystrophy as a result of compound heterozygous *CAPN3* mutations, and have muscle pathology findings that are not compatible with HMERF. The clinical picture was of juvenile onset proximal myopathy without respiratory muscle weakness, and thus also distinct from HMERF’s described clinical spectrum. Furthermore, sequencing of two unaffected family members from the same family revealed the presence of the *TTN* g.296459C>T/p.R32450W kinase variant in one of them. This is consistent with the kinase variant being a neutral polymorphism, and not the cause of disease in this family.

We agree with Hedberg *et al.* (2013) that mutations in the 119th fibronectin-3 domain of *TTN* cause HMERF, and that the p.R32450W kinase domain variant is not sufficient to cause the disease. The data presented here, from both patients and controls, provide no evidence to support the role of kinase domain mutations in HMERF, in keeping with public data listing the g.296459C>T/p.R32450W variant in the kinase domain of *TTN* as a single nucleotide polymorphism (rs140319117). Although currently also listed as pathogenic on the Human Gene Mutation Database (HGMD, CM057411), we suggest that g.296459C>T/p.R32450W should be removed from HGMD to avoid erroneous genetic counselling advice in the future.
